# 3D hierarchical porous hybrid nanostructure of carbon nanotubes and N-doped activated carbon

**DOI:** 10.1038/s41598-020-75831-x

**Published:** 2020-11-02

**Authors:** Piotr Kamedulski, Wojciech Zielinski, Pawel Nowak, Jerzy P. Lukaszewicz, Anna Ilnicka

**Affiliations:** 1 Faculty of Chemistry, Nicolaus Copernicus University in Torun, Gagarina 7, 87-100 Torun, Poland; 2 Centre for Modern Interdisciplinary Technologies, Nicolaus Copernicus University in Torun, Wilenska 4, 87-100 Torun, Poland

**Keywords:** Chemistry, Materials science, Nanoscience and technology

## Abstract

In this work, carbon nanotubes (CNTs)/nitrogen-doped activated carbon (AC) hybrids were designed and fabricated using a facile and one-step synthesis. The synthesis of CNTs is based on the recently discovered phenomenon of thermally-induced polyfurfuryl alcohol (PFA) conversion. Hybrid materials are fabricated through the in-situ free growth of closed carbon nanotubes on low-cost activated carbon substrates which were obtained from green algae or amino acids. Herein, three types of carbon nanotubes were observed to freely grow on an activated carbon background from *Chlorella vulgaris* or l-lysine, types such as multiwalled carbon and bamboo-like nanotubes, whose structure depends on the background used and conditions of the synthesis. Structure type is identified by analyzing transmission electron microscopy images. HRTEM images reveal the tubes’ outer diameter to be in the range of 20–120 nm. Because the carbon surface for the growth of carbon tubes contains nitrogen, the final hybrid materials also possess pyridinic-N and quaternary-N groups, as indicated by X-ray photoelectron spectra.

## Introduction

Carbon nanotubes (CNT), due to the high surface-to-weight ratio, have excellent electrochemical properties and high mechanical strength, which have made them a source of research curiosity among scientists and broad hopes for their practical applications. CNTs can be divided into two types, based on their crystalline structures: crystalline CNTs and amorphous CNTs. The main techniques for CNT growth are arcing, pyrolysis, thermal vapor deposition (CVD), and laser vaporization^1–4^. Traditional methods of CNT synthesis are complex procedures and based on specific experimental setups, like the presence of vacuum (or high pressure), high temperature, a plasma booster, and a well-selected catalyst, which would provide the right environment for spontaneous nanotube growth. Recently elaborated methods^5^ make it possible to replace the traditional ones and replace typical CNTs with an amorphous form of CNT in such a way that the efficiency of the procedure is high and the method can be transferred to an industrial scale. The new method was developed with the aim of synthesizing CNT-based electrode materials, hoping for effective maintenance or improvement of electrochemical properties.

Constantly growing interest in electrode materials has generated new paths for obtaining completely new substances from those previously known, such as carbon or its derivatives, as well as materials completely free of metals. In recent years, carbon nanotubes and their porous hybrids, which could become an alternative form to known precious metals such as platinum, ruthenium, or iridium, have enjoyed wide interest. Due to the variety of ways to obtain hybrid carbon materials with unique properties, the scope of their potential application is no less wide. Hybrid carbon nanomaterials possess extremely promising electrochemical properties^6^. In energy storage devices, such as supercapacitors^7–9^, lithium-ion batteries^10,11^, sodium-ion batteries^12^, and fuel cells^13^ they can be used as adsorbents for purifying water and removing harmful compounds and heavy metals^14–16^, as electrodes to remove NaCl from a saltwater solution^17^, as capacitive deionization electrodes^18,19^, and in composites, e.g., for the production of refractory materials^20^.

Composites of polyaniline (PANI) with carbon nanotubes (CNTs)^21^, graphene^22^, or ordered mesoporous carbon (OMC) material^23^ show high specific capacities and long-term stability when tested as electrode material in an electrochemical cell^24^. To overcome certain problems with material properties, some groups have recently reported preparing nanocomposites containing CNTs and ordered mesoporous carbon (OMC) by combining the advantages of both carbon materials. These OMC/CNTs nanocomposites, with a three-dimensionally (3D) interconnected pore structure, high specific surface area, and improved electronic coductivity, exhibited excellent electrochemical performance in areas of energy storage systems^25–28^. Their OMC mesostructure reduced ion transport resistance and ion diffusion distance for high-rate supercapacitors and enhanced charge storage^29–31^. In some cases graphene oxide was added to further improve the conductivity of CNT/ordered mesoporous carbon (OMC)^29^. Besides electrochemical applications, the CNT/OMC composite could be used as support for a g-C_3_N_4_ photocatalyst and displayed excellent catalytic performance in the photoreduction of CO_2_ with H_2_O to produce value-added fuels^32^.

The disadvantage of many methods of synthesizing hybrid carbon materials is the presence of heavy metals in the structure, as they lead to higher electrode costs and decrease the commercialization potential of energy storage devices. Jo et al. achieved CNT/ordered mesoporous carbon (OMC) via the nanocasting route using SBA-15-type, hexagonally-ordered mesoporous silica (OMS) as a template and nickel phthalocyanine (NiPc) as a precursor^33^. This work provides a way of structurally designing a low-cost, Pt-free, high-performance counter electrode material. One year later, Cheon et al. fabricated three-dimensional (3D) CNT/AC hybrid architectures via a chemical vapor deposition (CVD) process, where the open-tipped CNTs were grown on the surface of bamboo-based AC obtained from sucrose and phthalocyanine^13^. To improve the adsorption or electrochemical properties of CNTs, they should be converted to bulky materials, whose shapes are determined by their intended applications. One promising way is to combine or fix CNTs with activated carbon. As of now, several methods have been developed of preparing carbon nanotubes/activated carbon (CNTs/AC) composite spheres, including methods like a high-pressure reaction and carbonization^34^ process, or one consisting of only carbonization under argon flow, where phenolic resin is used as the carbon source^35^.

The introduction of nitrogen into hybrid nanostructures can improve their catalytic performance; following this, Zhao et al. synthesized a N-doped, AC-based hybrid nanostructure decorated with CNTs using a mechanical milling and pyrolysis approach^36^. Another way to prepare composite CNT/activated carbon consists of polymerization followed by high-temperature carbonization and steam activation^37,38^. Wang et al. synthesized CNT/carbon (CNT/C) composite spheres with radial laminar channels, from the center to the surface, however the as-produced spheres show a fragility attributed to the many large pores formed during freeze casting^39^. On the other hand, Guo et al. proposed a flexible and convenient oil-drop method of preparing porous CNT/AC composite spheres^40^. Bai et al. proposed a scalable extrusion-spheronization method followed by carbonization to fabricate CNT/C composite spheres. In this work, composite spheres are obtained by extruding a moistened mixture of CNTs and microcrystalline cellulose and subsequently spheronizing the broken extrudates^41^. Three-dimensional CNT/AC hybrid architectures can be fabricated by a chemical vapor deposition process using well-dispersed cobalt nanoparticles as growing seeds and a bamboo-based AC surface^9^. The electrochemical performance of a CNT/AC composite depends on the proper mass ratio—a mass ratio equal to 1 significantly improves the capacitance of a supercapacitor^7^. An electrode based on CNT/AC minimizes both electrical and ionic resistance of the electrolyte in the resulting carbon electrodes^42^. Using this type of composite, capacitance retention rose to 85% after 11,000 cycles, which implies that the carbon particles in the electrode material are strongly adhered to each other and that the electrode is stable over time^43^. An important finding was the possibility of fabricating N-doped CNT/AC electrodes where polypyrrole was the source of N-doped carbon, improving the electron transfer in supercapacitor electrodes at high current loads^8^. In this work, authors present the synthesis of CNT/N-doped AC where CNT growth on the AC surface is observed in one step of synthesis. The main difference of this procedure compared to other research is the absence of metal species in the carbon framework.

The current paper aims to demonstrate the synthesis of three types of carbon nanotubes: multiwalled carbon nanotubes, amorphous carbon rods, and bamboo-like nanotubes on N-doped activated carbon obtained from *Chlorella vulgaris* or l-lysine. CNTs/AC hybrids were designed and fabricated through the free growth of CNTs on an N-doped AC surface into a new type of hybrid. This study investigated the effect of carbonization temperature and reagent ratio on the shape and outer diameter of carbon tubes and the content of nitrogen groups on hybrid carbon materials. Hybrid materials combine the advantages of both N-doped AC and CNTs. The high specific surface area of CNTs/AC provides numerous metal-free, catalytically active sites containing nitrogen groups for future electrochemical applications.

## Results and discussion

**Figure 1:**
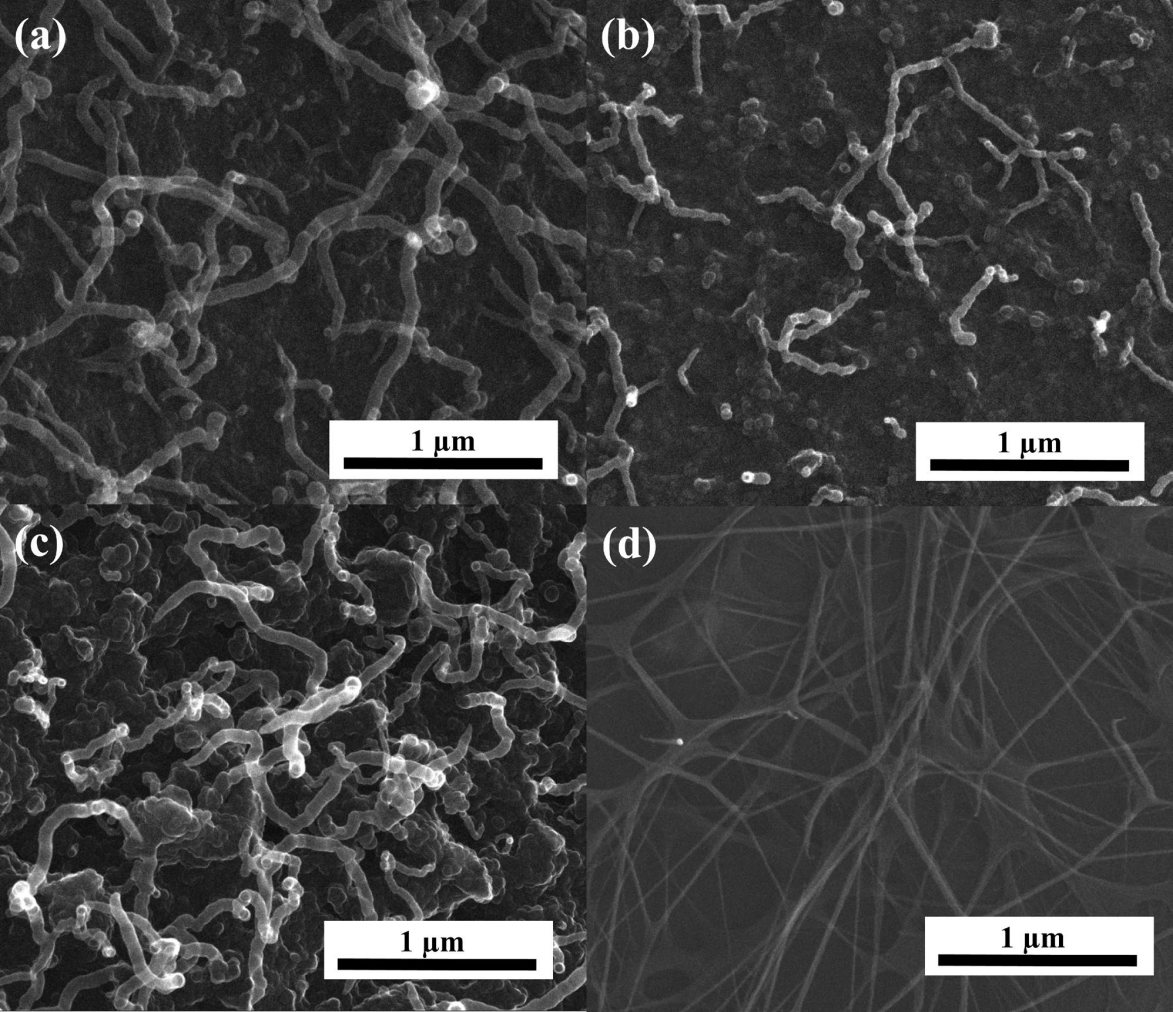
SEM image for samples (**a**) C1-A-7, (**b**, **c**) C2-A-7, and (**d**) C2-V-8.

**Figure 2:**
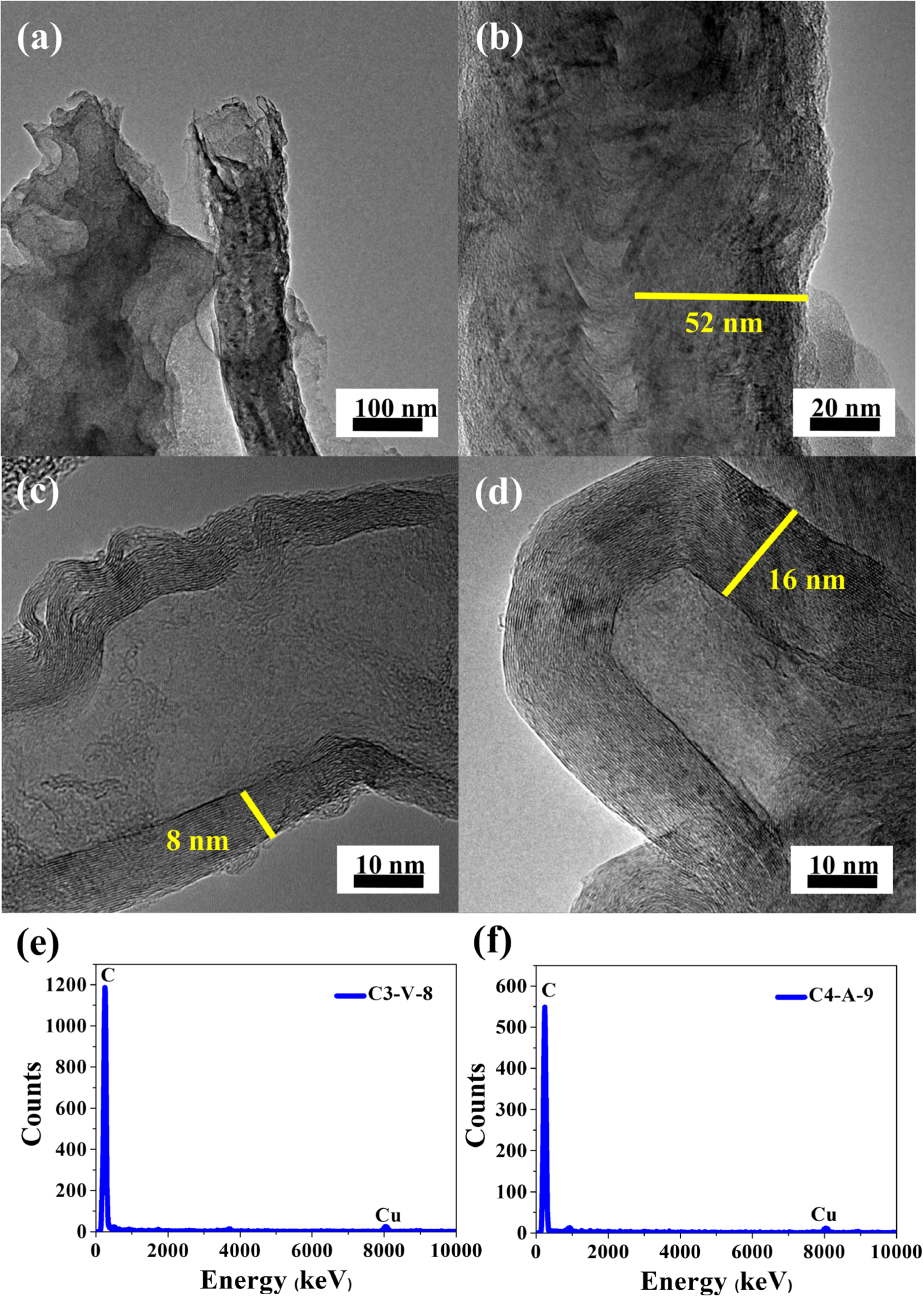
HRTEM images in different magnifications for samples (**a**, **b**) C2-A-7, (**c**) C3-V-8, and (**d**) C4-A-9, EDX elemental profiles for (**e**) C3-V-8, (**f**) C4-A-9.

CNT growth on N-doped AC surface was confirmed using instrumental methods. Extensive analysis can provide information on the new form of CNT/N-doped AC and give a new direction to the potential application of materials obtained with specific properties. The morphology of the CNT/N-doped AC hybrid carbon materials was determined based on SEM and HRTEM images. CNTs grown on the surface of N-doped carbon from *C. vulgaris* have different tube wall structures compared with crystalline CNTs grown on the background of N-doped carbon from l-lysine. Figure 1 shows the SEM images of CNTs/AC. The outer diameters of CNTs for samples C1-A-7, C2-A-7, and C2-V-8 are 37–64, 29–95, and 8.5–41 nm, respectively. These results confirm that for the higher carbonization temperature of 800 °C, the outer diameter of CNTs is smaller compared to samples carbonized at 700 °C. Figure 2 shows high-resolution transmission electron microscopy (HRTEM) images of a CNT/N-doped AC.

The HRTEM images presented in Fig. 2 show the presence of well-graphitized CNTs with an outer diameter of 20–120 nm open tip tilted from the vertical direction for C2-A-7 sample and with a closed tip for C3-V-8 and C4-A-9. The graphitic layers are clearly visible for all analyzed samples. The parallel graphene sheets of the C2-A-7 sample possess (Fig. 2a,b) perfect graphitic regions alternated with defective regions. The tube outer wall structure of the C2-A-7 sample consists of discontinuous graphene sheets and carbon clusters with short-range order and long-range disorder. The energy-dispersive X-ray spectroscopy (HRTEM-EDX) analysis in Fig. 2e,f indicates that the distribution of elements in the CNTs points to carbon as the main component. The content of carbon is equal to 100 at.%. Carbon in the aromatic rings plays a role in producing graphene layers in the CNTs structure, as was confirmed in these results. Small peaks are observed on the EDX profile for copper from the Cu mesh used as sample background.

**Figure 3:**
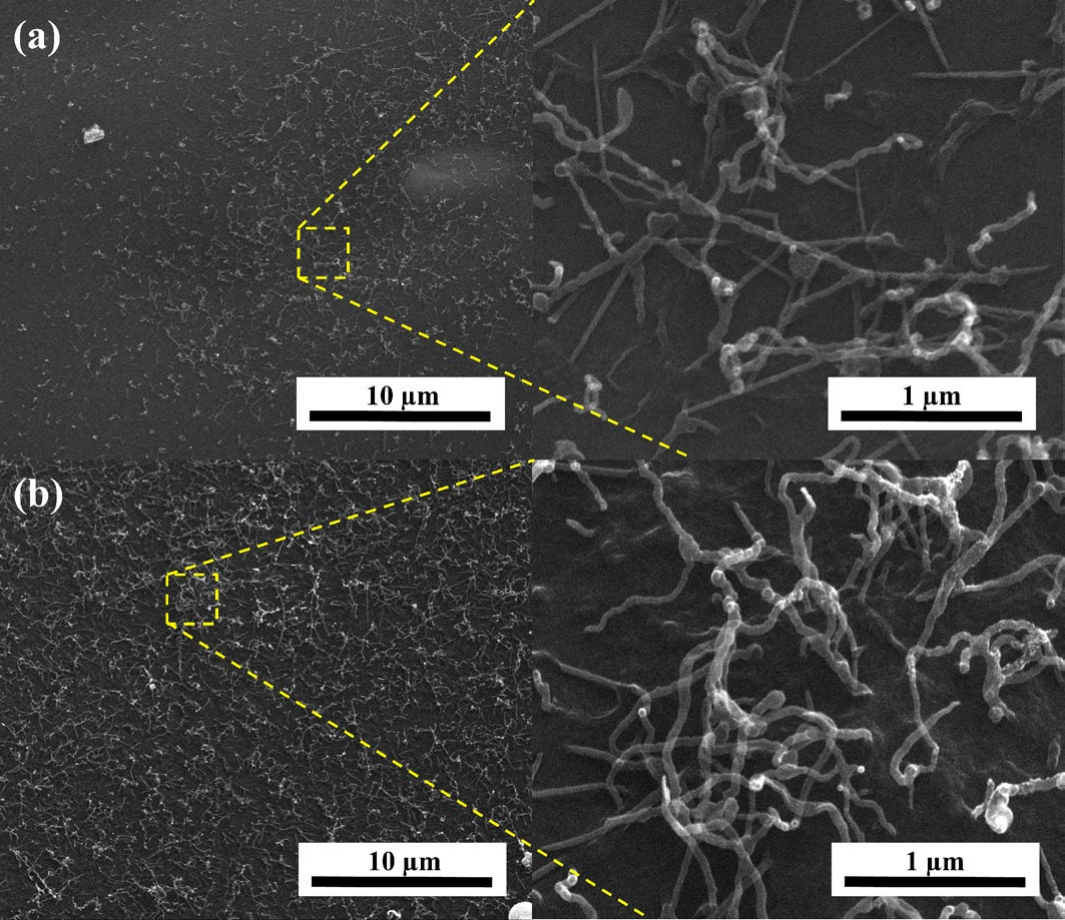
SEM images for samples (**a**) L2-A-7 and (**b**) L2-A-9 in different magnifications.

**Figure 4:**
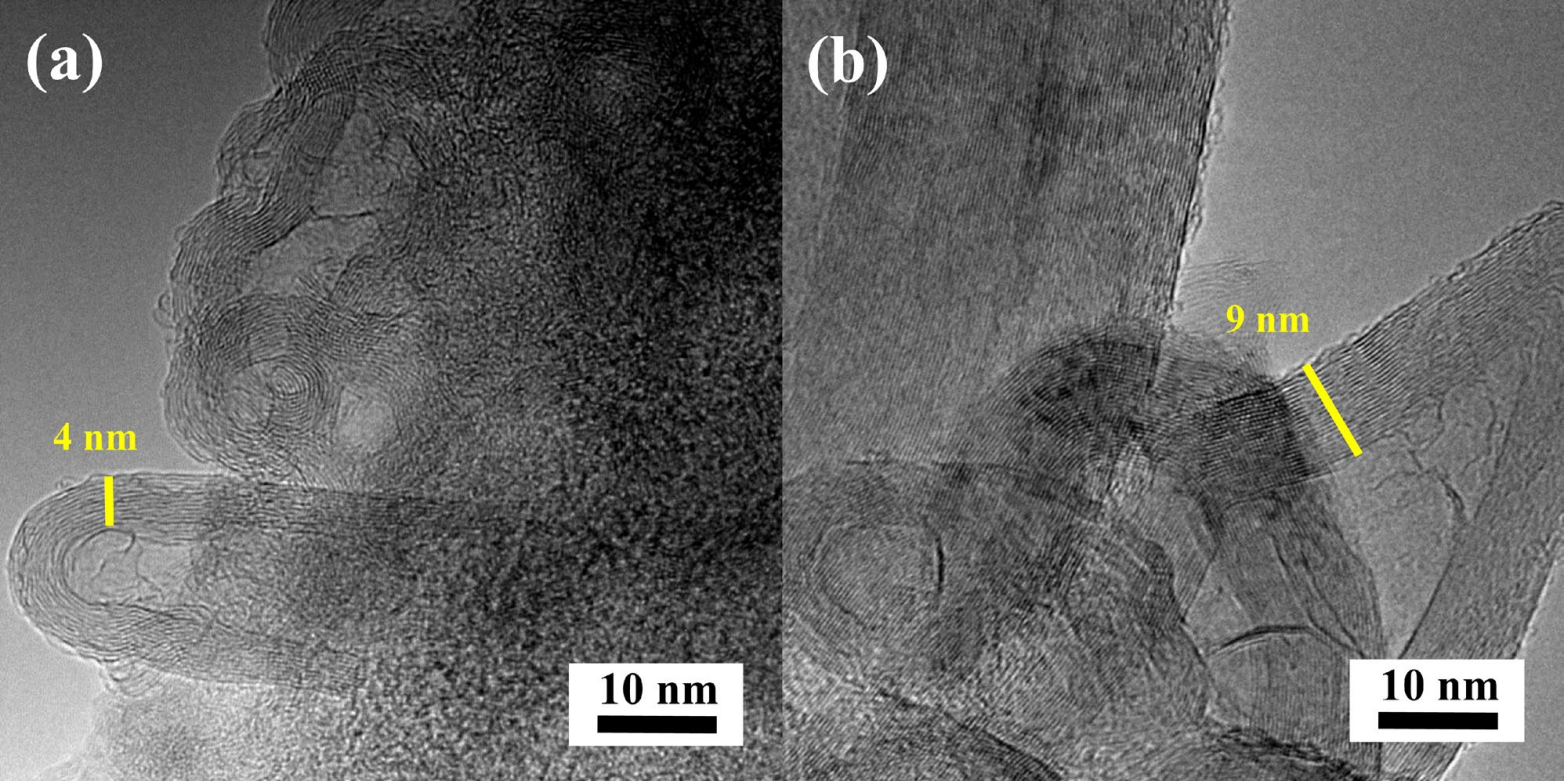
HRTEM images for samples (**a**) L2-V-9 and (**b**) L2-A-9.

**Figure 5:**
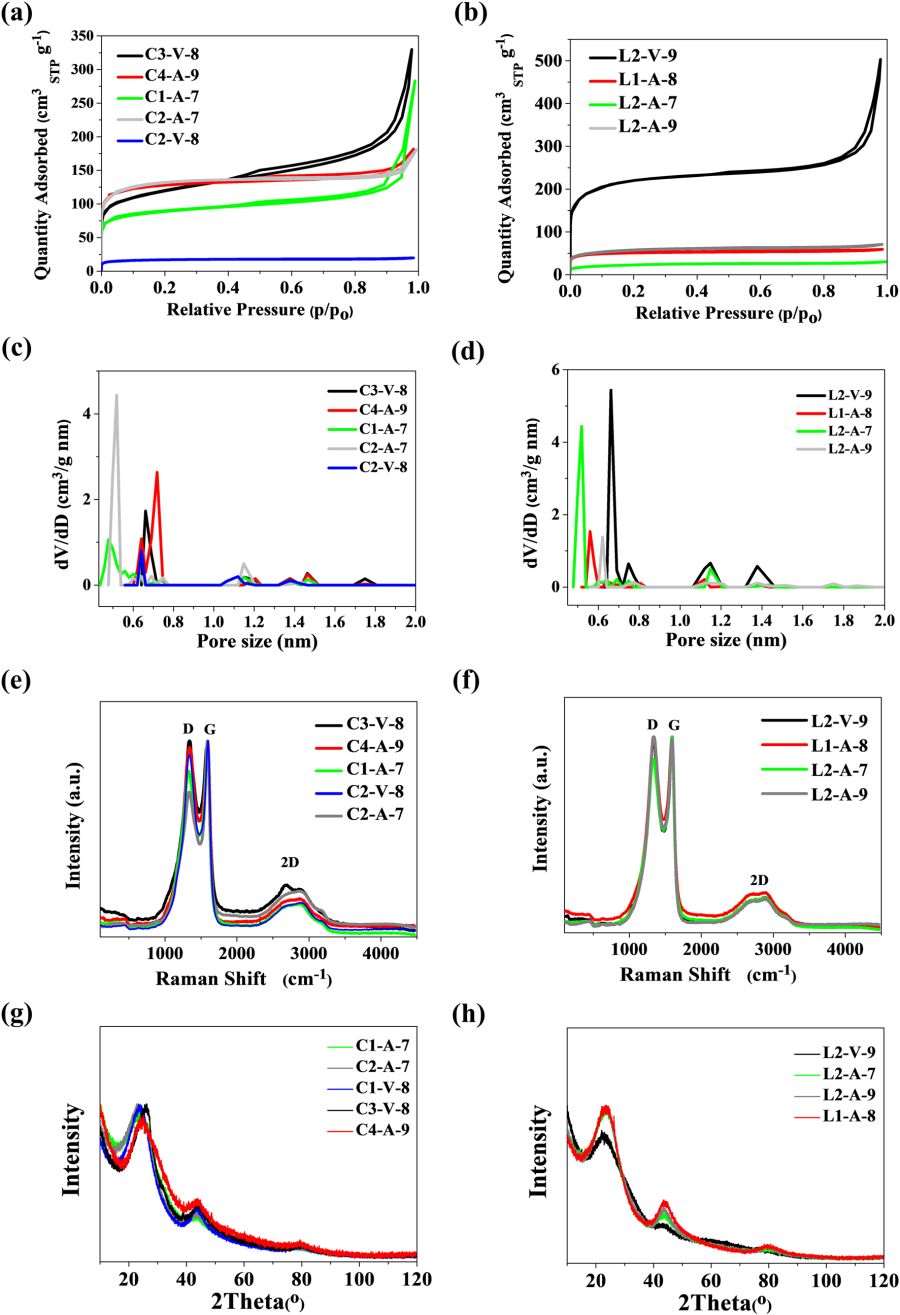
(**a**, **b**) Nitrogen adsorption–desorption isotherms; (**c**, **d**) pore size distribution determined by the NLDFT method; (**e**, **f**) the Raman spectra of the N-doped hybrid CNT-AC obtained on AC from *C. vulgaris* and l-lysine background; XRD patterns of (**g**) CNT/N-doped AC from *C. vulgaris*, (**h**) CNT/N-doped AC from l-lysine.

In contrast to the series of hybrid materials obtained on a N-doped carbon surface from *C. vulgaris*, the CNTs carbon nanostructure prepared with N-doped carbon from l-lysine showed clear differences in morphology. Figure 3 shows the SEM images of CNTs/N-doped AC hybrid carbon nanomaterials produced on a N-doped carbon l-lysine background. The outer diameter of tubes for the L2-A-7 and L2-A-9 samples was about 15–40 and 30–45 nm, respectively. The HRTEM images for this series of hybrid carbon materials (Fig. 4) confirm the presence of MWCNTs in the L2-V-9 and L2-A-9 samples, with an outer diameter in the range of 20–120 nm and dominant wall thickness in the range of 4–9 nm. Tubes and rods along the axial direction were not straight, but twisted in all places. The porous structure and surface area of the CNTs/N-doped AC hybrid materials were examined with nitrogen adsorption and desorption isotherms (Fig. 5a,b). The specific surface area was measured using the Brunauer–Emmett–Teller (BET) method. The highest specific BET surface areas in the series CNT/N-doped AC from *C. vulgaris* were those of samples C4-A-9 and C2-A-7, at 451 m^2^ g^−1^ and 499 m^2^ g^−1^, respectively. The BET analysis revealed that sample L2-V-9 had the largest surface area, 827 m^2^ g^−1^. The series CNT/N-doped AC from l-lysine had a very large specific surface area, while that CNT obtained on N-doped AC from *C. vulgaris* was the smallest. Furthermore, the small hysteresis loop at the relative pressure of 0.4–1.0 suggests that the hybrid materials have a microporous structure. The pore size distributions of each sample are shown in Fig. 5b,c. Low temperature nitrogen adsorption–desorption isotherms were determined experimentally for CNTs/N-doped AC hybrids. The isotherms may be accounted to the I type according to the IUPAC classification.

Based on the nonlocalized density functional theory (NLDFT) method, the pore size distributions (Fig. 5c,d) for all obtained materials are in the range of 0.4–0.8 nm with small peaks at about 1.2 and 1.4 nm. As elemental composition is strongly dependent on manufacturing conditions, a CHN combustion analysis was performed to collect information on the presence of two major elements, i.e., carbon and nitrogen. The carbon content was in the range of 66–86 wt.% for all samples. The range of nitrogen content was between 0.30 and 2.74 wt.%. The content of nitrogen depended on the carbon background (C1 or C2). The samples’ graphitization degree can be studied with Raman spectra. The analysis of Raman spectra was based on three basic bands, i.e., D, G and 2D, which were clearly visible. The peak at about 1340 cm^−1^ (D-band) suggests the presence of surface defects (sp^3^ hybridized carbon) and is often attributed to the disordered structure of graphite. The peak at about 1590 cm^−1^ (G-band) confirms well-crystallized graphite carbon (sp^2^ hybridized carbon)^16,44^. The G band is attributed to E_2g_, a stretching vibration mode of graphite, and the D band corresponds to A_1g_, a breathing vibration mode of disordered graphite, which suggests the presence of defects within the hexagonal graphitic layers^45^. The I_D_/I_G_ ratio of the CNTs is near below 1. It shows that this nanotube has a very low graphite crystallinity and many defects within its structure. This is in agreement with the results in Fig. 5e,f. Based on Raman spectroscopy data, no single-wall tubes were observed and only multiwalled CNTs were present.

After the growth of CNTs on the N-doped AC surface, the D- to G-band intensity ratio (I_D_/I_G_) for the CNT/N-doped AC sample increased slightly with the rising carbonization temperature, suggesting an increase in the degree of graphitization. The I_D_/I_G_ ratios of L2-V-9 and L1-A-8 were equal 1.01 and 0.99, respectively, suggesting that L2-V-9 had a slightly higher degree of graphitization than L1-A-8, because the bamboo-like N-doped CNTs had a higher degree of graphitization than the amorphous carbon. XRD patterns of CNT/N-doped activated carbon obtained from *C. vulgaris* and l-lysine are shown in Fig. 5g,h. The crystal plane diffraction peak of hybrid carbon materials appears at ~ 23° (002 reflection of graphitic carbon) and a small peak at ~ 43° (100 reflection of graphitic carbon) were determined. The shape of these peaks is not sharp, as final hybrid carbon materials contain not only CNTs, but also N-doped amorphous activated carbon used as a background for CNTs growth.

**Figure 6:**
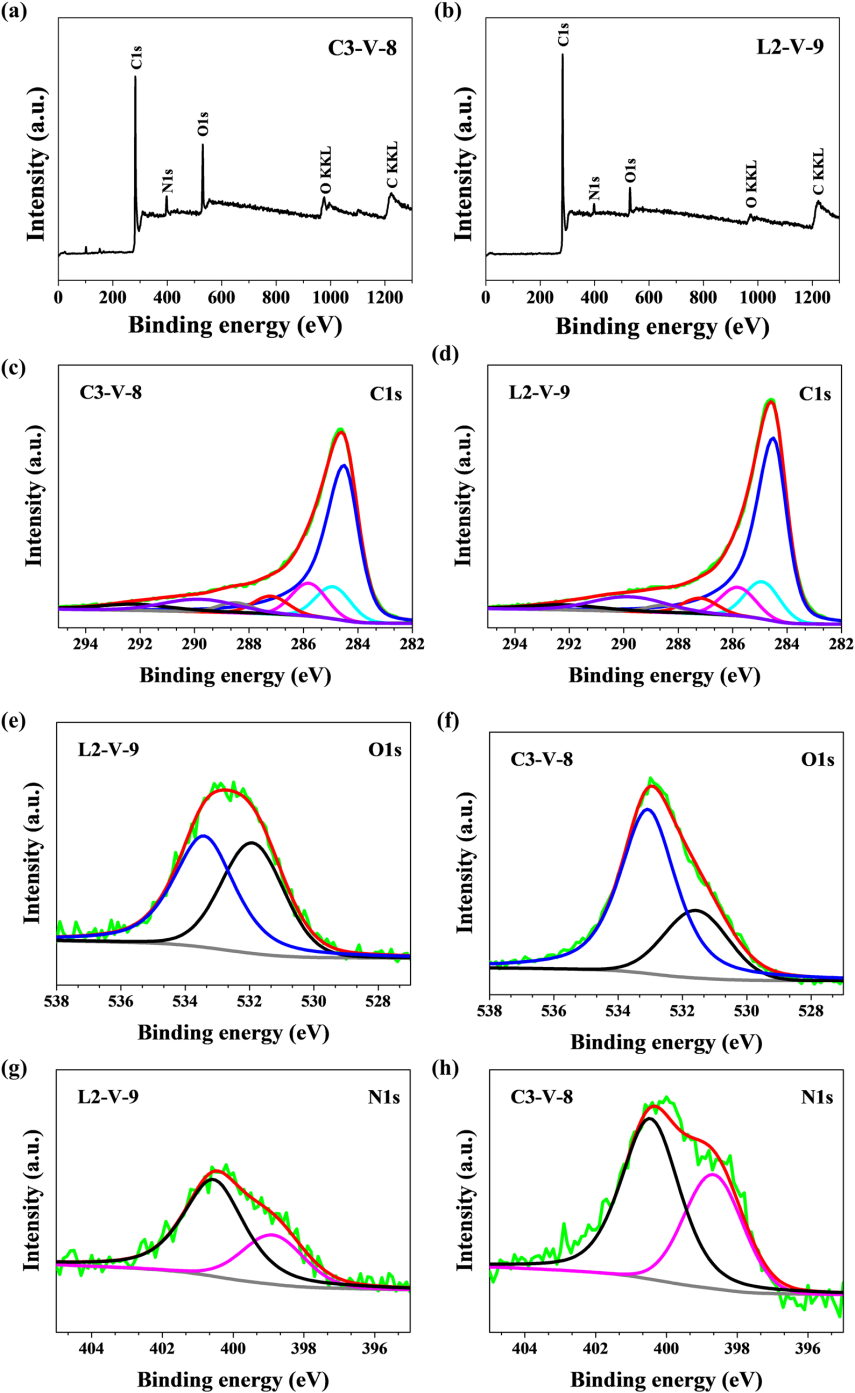
(**a**, **b**) XPS survey spectra and high-resolution X-ray photoelectron spectra for C1s, N1s, and O1s of (**c**, **e**, **g**) C3-V-8, (**d**, **f**, **h**) L2-V-9 sample.

X-ray photoelectron spectroscopy can provide information about the chemical structure of CNT/N-doped AC. The content of the three main components in CNT/N-doped AC are shown in Table 1. The XPS spectra of CNT/N-doped AC are demonstrated in Fig. 6. The following sub-peaks are visible after the deconvolution of the general peak C1s: C=C bond (sp^2^) peak at 284.6 eV^46^, C–C bond (sp^3^) peak at 285 eV^46,47^, C–O–C or C–OH or C–NH bond peak at 286.3 eV^46,47^, C=O or O–C–O or N–C–O bond peak at 287.7 eV^46,47^, O–C=O peak at 288.6 eV^46^, and peaks with binding energy at 289.6 eV and 292.1 eV are associated with shake-up excitation^48^. Shake-up excitation is derived from sp^2^ carbon and its aromatic forms, and is an additional parameter confirming the presence of this type of bond^46,49^. The analysis of O1s spectra revealed content of elemental oxygen in the range of 5.6–12.7 at.%. The deconvolution of the O1s peak reveals different states of oxygen, whose peak at 532.0 eV signifies the presence of an O=C–N or C=O bond and the peak at 533.3 eV is characteristic of an O*=C–O or O–C–O bond^46,47,49^. The high-resolution N1s spectra (Fig. 6b,e) can be deconvoluted into two peaks, located at 400.5 and 398.7 eV, which are attributed to quaternary (N-Q) and pyridinic nitrogen (N-6) groups^46,47^, respectively. It is known that the N-Q and N-6 groups have a stronger donor electron character, thus improving the electron transfer in supercapacitor electrodes at high current loads^50^. In addition, N-Q and N-5 nitrogen located at the edges of graphene layers enhance the pseudocapacitance effect, wettability, and hydrophilicity of the electrode^51^. As shown in Fig. 6, L2-V-9 had pyridinic N lower than that of C3-V-8, while C4-A-9 lacked pyridinic N; C3-V-8 had quaternary N groups levels higher than those of C4-A-9. A weak P2p peak in the C4-A-9 spectrum was observed because of H_3_PO_4_ being used as a catalyst for FA polymerization. The P2p spectrum with the 2p_3/2_ main line at 133.4 eV binding energy indicates the presence of phosphorus in PO_4_^3−^ tertiary phosphate or secondary HPO_4_^2−^.

**Table 1:** Chemical composition analyzed by XPS for N-doped hybrid CNT-AC obtained on AC from *C. vulgaris* and l-lysine background.

Sample	Binding energy (eV)															
	284.6	285.0		286.3		287.7		288.6		532.0		533.3		398.7		400.5
**Elemental content (at.%)**																
C3-V-8	57.8	7.8	7.9		4.3		2.4		3.4		9.3		2		3.3	
C4-A-9	65.8	10.5	7.4		2.8		0.8		4.2		5.2		0		1.2	
C1-A-7	25.8	33.5	13.4		4.6		3		3.7		7.7		3.3		2.6	
C2-A-7	26.3	41.8	5.8		4		1.1		6		10.1		0.4		0.1	
C2-V-8	32.8	41.2	7.2		4.3		1.2		3.8		6.3		0.3		0.1	
L2-V-9	67.7	9.7	7.5		4.1		1.9		2.5		3.1		1		2.4	
L1-A-8	26.9	36.6	12.4		4.3		3.2		4.2		6.7		1.8		1.2	
L2-A-7	28.7	36.5	9.1		3.9		2.1		6.3		9.2		0.3		0.1	
L2-A-9	34.5	37.9	8.3		4.1		1.4		3.7		6.7		0.3		0.1

## Conclusions

In this paper, CNT/N-doped AC hybrid materials were successfully synthesized through free growth due to the direct thermal conversion of a polymer (PFA). The present work could provide a new pathway to design and develop novel types of N-doped carbon using a low-cost, metal-free, and eco-friendly method. Morphological studies were performed using high-resolution scanning electron microscopy (SEM) and high-resolution transmission electron microscopy (HRTEM). The studies made possible to determine the size and shape of the carbon nanotubes produced on the N-doped AC surface. Most tubes are multiwalled with a diversity of inner and outer diameters, up to 72 nm and 120 nm, respectively. Besides typical crystalline ones, amorphous tubes grew on the carbon background surface. In general, the CNTs and bamboo-like structure formed closed tubes on the micro or micro-mesoporous carbon surface. The hybrids exhibited not only a high surface area of up to 827 m^2^ g^−1^ and a high degree of graphitization, but also high contents of both pyridinic- and quaternary-N groups. Crystalline and amorphous carbon nanotubes with an outer diameter up to 120 nm were observed to grow on the nitrogen-doped porous carbon surface. The structural property results of CNTs/N-doped AC hybrid carbons confirm that the hierarchical carbon nanostructures comprising 1D CNTs and 3D porous carbons are promising materials in energy storage devices. Their structural properties will facilitate the high-rate transportation of electrolyte ions and electrons throughout the hybrid carbon electrode matrix, with a potential high specific capacitance.

## Materials and methods

### Preparation of CNT/N-doped AC from *C. vulgaris*

The N-doped mesoporous activated carbon from *C. vulgaris* was synthesized following a previously described method^44^]. Two series were selected for research, depending on the mass ratio in which calcium carbonate (size of particles 15–40 nm) was added to *C. vulgaris* powder—4:3 or 1:1. Distilled water was added and the resulting slurry was homogenized by means of an electric blender, then dried to evaporate excess water. This mass was heated in a tube furnace (Thermolyne F21100) at a rate of 10 °C min^−1^, under the flow of highly pure N_2_, up to the temperatures of 800 and 900 °C. It was kept in these conditions for 1 h, and was cooled to room temperature afterwards. The resulting materials were treated with a HCl aqueous solution (36 wt.%) and rinsed with distilled water on a Büchner funnel until neutral pH was reached. The received carbon materials of both series were dried in an electric oven at 105 °C overnight. Each sample is denoted by a unique symbol consisting of letters and digits, CL_X_T, where: CL—*C. vulgaris*, X—template CaCO_3_ used in a mass ratio 4:3 (C) or 1:1 (D), T—carbonization temperature (°C). In this research CL_D_800, CL_D_900, CL_C_800, and CL_C_800 samples were used, denoted further as C1, C2, C3, and C4, respectively. 0.28 g of the obtained C1, C2, C3 or C4 N-doped activated carbon was treated with a volume ratio of (furan-2-yl)methanol : H_3_PO_4_ (75 wt.%) equal 3:1. The modified sample was put into the oven in air (signed as A) or vacuum (signed as V) conditions at 200 °C for 1 h. After that, the mass was carbonized at a rate of 10 °C min^−1^, under the flow of pure N_2_, to the temperature of 700, 800, or 900 °C for 1 h, then cooled to room temperature. Carbonization temperature was denoted in the sample name as 7, 8, or 9, respectively.

### Preparation of CNT/N-doped AC from l-lysine

Authors have extensive experience in the synthesis of N-doped activated carbons based on different natural and N-rich precursors^52–54^. Among natural precursors, l-lysine was found to be a promising candidate for carbonization, which in parallel provides advanced structural parameters (specific surface area, pore structure) and a high nitrogen content^55^. Therefore, in this study too was l-lysine selected as a suitable precursor to manufacturing bases for subsequent of CNT growth (new method). The N-doped, mesoporous activated carbon from l-lysine was synthesized following a previously described method^55^. In the synthesis procedure, l-lysine powder was added to particle CaCO_3_ (particle size 15–40 nm) and stirred mechanically. Then, distilled water was added to the mixture, and the mass was kept at room temperature for 1 h while stirred magnetically. To evaporate the distilled water, the mass was dried in an electric furnace at 80 °C for 24 h. In the next step, the dry combination of l-lysine and CaCO_3_ was heated up under the flow of highly pure N_2_ at a rate of 10 °C min^−1^, until it reached temperatures of 800 and 900 °C; it was held at this temperature in a tube furnace for 1 h (Thermolyne F21100), then cooled to room temperature. Next, it was treated with an HCl aqueous solution (36 wt.%) and subsequently rinsed with distilled water on a Büchner funnel until neutral pH was achieved. The resulting carbon substances were dried in an electric oven at 105 °C overnight. Further in the text, N-CFO-T is used as a unique denotation, where T is the carbonization temperature (°C). In this research, N-CFO-800 and N-CFO-900 samples were used, denoted further as L1 and L2, respectively. The produced 0.1 g of L1 or L2 N-doped activated carbon was treated with a volume ratio of (furan-2-yl)methanol:H_3_PO_4_ (75 wt.%) equal 3:1. The modified sample was then placed in the oven in air (signed as A) or vacuum (signed as V) conditions at 200 °C for 1 h. Then, the mass was carbonized at a rate of 10 °C min^−1^, under the flow of pure N_2_, to the temperatures of 700, 800, or 900 °C for 1 h, then cooled to room temperature. Carbonization temperature was denoted in the sample name as 7, 8, or 9, respectively.

### Characterization

Scanning electron microscopy (SEM) was performed using a SEM 1430 VP instrument (LEO Electron Microscopy Ltd., Germany). A high-resolution transmission electron microscope (HRTEM) with an energy dispersive X-ray spectrometer (EDX) was used to determine atomic structure (FEI Tecnai F20 X-Twin microscope, Czech Republic). The textural properties and nitrogen sorption isotherms of CNT/N-doped AC were determined by nitrogen physisorption experiments at − 196 °C using an ASAP 2020 Plus instrument (Micromeritics, USA). The apparent surface area was calculated by the Brunauer–Emmett–Teller (BET) equation. The pore size distributions were determined by means of the non-local density functional theory (NLDFT) method. The elemental composition was analyzed by a Vario CHN analyzer (Elementar Analysensysteme GmbH, Germany). X-ray photoelectron spectroscopy (XPS) analysis was conducted with Al Kα radiation (PHI5000 VersaProbe II Scanning XPS Microprobe, Japan). The survey spectra were recorded in the energy range of 0–1300 eV. Raman spectra were recorded with a Renishaw InVia Raman analyzer with an excitation wavelength of 532 nm (Renishaw Company, UK). X-ray diffraction (XRD) patterns were used to identify the phase structure of CNT/N-doped AC (X’Pert-Pro, Philips, Cambridge, UK) with Cu Kα radiation.
